# Association of leptin, toll-like receptor 4, and chemokine receptor of interleukin 8 C-X-C motif single nucleotide polymorphisms with fertility traits in Czech Fleckvieh cattle

**DOI:** 10.5713/ajas.17.0900

**Published:** 2018-04-12

**Authors:** Katerina Jecminkova, Uwe Müller, Jitka Kyselova, Zuzana Sztankoova, Ludmila Zavadilova, Miloslava Stipkova, Ivan Majzlik

**Affiliations:** 1 Institute of Animal Science, Prague 10400, Czech Republic; 2 Faculty of Agrobiology Food and Natural Resources, Czech University of Life Sciences Prague, Prague 16500, Czech Republic; 3 Albrecht Daniel Thaer-Institute of Agricultural and Horticultural Sciences, Humboldt-Universität zu Berlin, Berlin 100 99, Germany

**Keywords:** Polymorphism, Reproduction, Leptin, Toll-like Receptor 4, Chemokine Receptor of Interleukin 8 C-X-C Motif, Cow

## Abstract

**Objective:**

The use of genetic markers can help to enhance reproduction in cattle, which is a very important trait for profitability in dairy production systems. This study evaluated the association between genotypes of leptin (*LEP*), toll-like receptor 4 (*TLR4*), and chemokine receptor of interleukin 8 C-X-C motif (*CXCR1*) genes and fertility traits in Czech Fleckvieh cattle.

**Methods:**

Phenotypic data from 786 Czech Fleckvieh cows raised on 5 farms in the Czech Republic were used, along with information from the 1st three parities. To determine genotype, the polymerase chain reaction– restriction fragment length polymorphism method was used.

**Results:**

Except for *LEP* g.-963C>T, all studied genotype frequencies of single nucleotide polymorphisms (SNPs) were distributed according to the Hardy-Weinberg equilibrium. Two *LEP* SNPs (g.-963C>T and c.357C>T) were associated with the age at the 1st calving, days open (DO), pregnancy rate after 1st service (PR), and calving interval (CLI). In *LEP* g.-963C>T the TT genotype heifers firstly calved 24 days earlier than CC genotype and the CT genotype cow showed a tendency for shorter DO and higher PR. In *LEP* c.357C>T we observed longer CLI and DO period in TT cows. In general, we can propose the TT genotype of g.-963C>T as favorable and the TT genotype of c.357C>T as unfavorable for a cow’s fertility. Heterozygotes in *TLR4* c.-226C>G were significantly associated with shorter CLI, and presented a nonsignificant tendency to be associated with higher PR. In *CXCR1* c.777 C>G, we did not observe any relationship of this SNP with reproduction.

**Conclusion:**

Overall, the results showed that *LEP* could be an effective marker for improving reproduction in Czech Fleckvieh cattle. This study also provides novel insights into the relationship between *TLR4* and *CXCR1* SNPs and reproduction in dual-purpose cattle.

## INTRODUCTION

Fertility traits are important selection targets; however, they have low heritability, so accurate selection and genetic gain in fertility traits is a slow process. An important candidate gene for improving reproductive performance is leptin (*LEP*). In cattle, the *LEP* gene is mapped to chromosome 4, and its genetic variants have been associated mainly with milk yield [1], energy balance, and feed intake [2], and fertility [3].

The g.-963C>T mutation in the *LEP* promoter was firstly described in 2005 [2], and its association was confirmed with reproductive traits in cattle [3,4]. Single nucleotide polymorphism (SNP) c.357C>T is located on exon 3 and causes an amino acid change from Alanine to Valine [5]; it is also associated with age at 1st service and milk yield during 1st and 2nd lactations [3], somatic cell count, and milk composition [6].

Toll-like receptor 4 (*TLR4*) is located on chromosome 8. The transversion C>G in the 5′-upstream promoter region, known as g.-226C>G, alters the expression of *TLR4* [7]. *TLR4* encodes an innate immune protein on cell surfaces and recognizes lipopolysaccharides of gram-negative bacteria [8]. There are numerous studies showing a higher expression of *TLR4* during the postpartum period and infection in Holstein cattle [9,10]. Early life immune activation via *TLR* signaling could have significant consequences for the programming of ovarian development and fertility in rats [11]. The role of *TLR* in reproduction was investigated and described in association of the *TLR* family and two variants of *TLR4* with brucellosis susceptibility [12].

Chemokine receptor of interleukin 8 C-X-C motif (*CXCR1*) is expressed on the surface of neutrophils and is associated with the inflammatory response to gram-negative bacterial infections [13]. In cattle, the *CXCR1* has been mapped to chromosome 2. The non-synonymous mutation, known as c.777C>G, substitutes Glutamine for Histidine in amino acid position 245 [14]. *CXCR1* has been associated with somatic cell count, milk yield, subclinical and clinical mastitis [15–17]. Therefore, this gene is suggested as a potential candidate marker for mastitis [14]. The level of interleukin 8 is in literature connected with reproduction disorders. Therefore, examination of its receptor in respect to the fertility traits could bring new results. The *CXCR1* c.777C>G was investigated also in relation to the reproduction diseases, but to date, no significant association has been found [16].

Czech Fleckvieh cattle are the original dual-purpose breed from central Europe, and are related to Montbéliarde, Fleckvieh, and Simmental breeds. As we know that among breeds and more over production types, the association can differ. It is therefore important to bring this information to light. However, neither the genotype frequencies nor the associations of studied genes are currently known for this breed. However, the identification of minor breeds and evaluation of marker assisted selection programs could also be useful for conservation strategies in gene resources breeds. Firstly, it is necessary to estimate the effects of chosen candidate gene markers in the local populations. The present study aimed to investigate the association between *LEP*, *TLR4*, and *CXCR1* SNPs and fertility traits. It also aimed to describe its genotype frequencies in Czech Fleckvieh cattle.

## MATERIALS AND METHODS

This study included 786 cows that descended from 27 bulls, that calved between 2003 and 2010 and were raised at 5 farms in the Czech Republic. Only the 1st three parturition were studied. Phenotypic data were obtained from the official progeny-testing. DNA was extracted from peripheral blood using ABI PRISM 6 100 Nucleic Acid PrepStation Instrument with BloodPrep reagents (Applied Biosystems, Foster City, CA, USA) following the manufacturer protocols. Blood was collected by veterinarian from the caudal vein in concordance with European and Czech laws.

Four SNP markers were genotyped using polymerase chain reaction–restriction fragment length polymorphism methods. These SNPs were genotyped according to mentioned authors and located in the promoter g.-963C>T; rs109956567 [18]) and exon 3 (c.357C>T; A59V; rs29004508 [5]) regions of the *LEP* gene, the promoter region of the *TLR4* gene (g.-226C>G; rs29017188 [19]), and in exon 1 of the *CXCR1* gene (c.777C>G; rs208795699 [20]).

GenAlex software [21,22] was used to determine allele and genotype frequencies, linkage disequilibrium between pairwise genotype combinations in *LEP*, and deviations from the Hardy-Weinberg (H-W) equilibrium.

Because the c.357C>T and c.777C>G are non-synonymous mutations, the *in silico* analysis was applied to predict functional impact of this SNPs (PROVEAN; http://provean.jcvi.org/index.php [23]), to estimate the likelihood that the SNP will have a functional impact on the protein (PANTHER; www.pantherdb.org/tools/csnp [24]) and to investigate the mutant protein stability (I-Mutant2.0; http://folding.biofold.org/cgi-bin/i-mutant2.0 [25]). Amino acid sequence gained from National Center for Biotechnology Information database in FASTA format was uploaded.

Association analyses were performed for age at 1st calving (AFC), period from calving to the 1st service (CFI), days open (DO), calving interval (CLI) and pregnancy after the 1st service (PR). Descriptive statistics for reproductive traits are shown in Table 1. The CLI at the 2nd parity is the interval between the 1st and 2nd calving, and interval between 2nd and 3rd calving is the CLI at the 3rd parity. Due to the considerable deviations from the normal distribution, all dependent variables (AFC, CFI, DO, CLI) were log-transformed for association analyses using Proc MIXED. In addition, residuals were checked for homogeneity. Cows were divided into 3 groups according to AFC; cows within a range of mean±1 standard deviation (SD) AFC were designated as the 1st group, the 2nd group consisted of cows that calved >(mean+1 SD), and the 3rd group consisted of cows that calved <(mean−1 SD). Except PR data were analyzed using the MIXED procedure of SAS 9.4. PR were class variables (1 = yes/0 = no), therefore the GLIMMIX procedure with a binomial distribution was used (SAS Institute Inc., Cary, NC, USA).

The following linear mixed models were used for statistical analysis:

- Repeated measurements for CFI, CLI, and DO:
  $${\text{Y}}_{\text{ijklm}}=\mu +{\text{HYS}}_{\text{i}}+{\text{Sire}}_{\text{j}}+\text{Age}\_\text{group}{\left(\text{Lac}\right)}_{\text{k}}+{\text{Lac}}_{\text{l}}+{\text{SNP}}_{\text{m}}+{\text{e}}_{\text{ijklm}}$$
- By parity measurement for CFI, CLI, and DO:
  $${\text{Y}}_{\text{ijkm}}=\mu +{\text{HYS}}_{\text{i}}+{\text{Sire}}_{\text{j}}+\text{Age}\_{\text{group}}_{\text{k}}+{\text{SNP}}_{\text{m}}+{\text{e}}_{\text{ijklm}}$$
- AFC:
  $${\text{Y}}_{\text{ijm}}=\mu +{\text{HYS}}_{\text{i}}+{\text{Sire}}_{\text{j}}+{\text{SNP}}_{\text{m}}+{\text{e}}_{\text{ijm}}$$
- Logistic regression with repeated measurements for PR:
  $${\text{P}}_{\left(\text{PR}\right)}=\text{a}+{\text{b}}_{1}{\text{HYS}}_{\text{i}}+{\text{b}}_{2}{\text{Sire}}_{\text{j}}+{\text{b}}_{3}\text{Age}\_\text{group}{\left(\text{Lac}\right)}_{\text{k}}+{\text{b}}_{4}{\text{Lac}}_{\text{l}}+{\text{b}}_{5}{\text{SNP}}_{\text{m}}$$
- Logistic regression by parity for PR:
  $$\text{P}(\text{PR})=\text{a}+{\text{b}}_{1}{\text{HYS}}_{\text{i}}+{\text{b}}_{2}{\text{Sire}}_{\text{j}}+{\text{b}}_{3}\text{Age}\_{\text{group}}_{\text{k}}+{\text{b}}_{4}{\text{SNP}}_{\text{m}}$$

Where, Y = observed trait; P_(PR)_ = probability of pregnancy after the 1st service; μ= population mean; a = intercept; HYS_i_ = combined fixed effect of the *i*th farm – year – season of calving (i = 1 to 17) in c) combined fixed effect of the *i*th farm – year – season of birth (i = 1 to 16); Sire_j_ = *j*th effect of cow’s father: in models a), d), e) fixed effect, in models b), c), random effect (j = 1 to 27); Age_group_k_ = fixed effect of kth group of AFC (k = 3); Lac_l_ = fixed effect of *l*th number of parity (l = 1 to 3); SNP_m_ = fixed effect *m*th genotype of the SNP (m = 1 to 3); b = corresponding regression coefficient of HYS, Sire, Age group, Parity, and SNP, respectively, e = vector of random residuals.

To choose the best covariance structure we used the Akaike Information Criterion (AIC). The model with the lowest AIC had the best fit and was utilized for analysis. To account for multiple testing, the p-values of the comparisons between genotypes were corrected using a Bonferroni’s adjustment. In context of the traits of interest and sample size, we report statistically nonsignificant tendencies with an alpha between 0.05 and 0.1. As the Proc MIXED cannot back-transform logarithmic values to standard error (SE), the 95% confidence intervals were calculated. For the GLIMMIX procedure, the SE was calculated.

We present results for repeated measurements and observations by parity to show how the associations differed in cows of different lactations and stages of physiological developments.

## RESULTS

### Genotype frequencies and linkage disequilibrium

Allelic and genotypic frequencies are presented in Table 2. The only locus showing a departure from H-W equilibrium was *LEP* g.-963C>T (p = 0.005); all other genotype frequencies of SNPs were distributed according to H W. In addition, there was linkage equilibrium between investigated *LEP* SNPs (D = 0.046) at p = 0.001. Therefore, we investigated each SNP separately.

### *In silico* analysis

The impact of an amino acid substitution predicted by PROVEAN software confirmed the c.357C>T and the c.777C>G SNPs as neutral. All the scoring from the *in silico* analysis is mentioned in Supplementary Table S1. In case that the score of the variant would be <−2.5 the variant should be considered as deleterious. According to PANTHER software the c.357C>T in *LEP* has been preserved in the nucleotide sequence for 220 million years (MY) and therefore is marked to be possibly damaging (450 MY>time>200 MY). The c.777C>G in *CXCR1* was revealed not to occur for a long time in the protein sequence and the direct ancestors were determined to appear 1 MY ago. Due to the short time, this mutation is considered as probably benign (time <200 MY).

Finally, we predicted the protein stability changes upon single-site mutation with reliability index at pH 7.0 and temperature of 38°C (average cow’s body temperature). In *LEP* c.357C>T the change from Alanine to Valine resulted in increased stability of the protein and in *CXCR1* c.777C>G was the stability decreased when the Glutamine was substituted by Histidine.

### Single nucleotide polymorphism association

Associations between SNPs of *LEP* and *TLR4* genes with fertility traits are presented for repeated measurement (Table 3) and according to parity number (Supplementary Figure S1–S4). For repeated measurement, we observed an association of *LEP* g.-963C>T SNP with AFC. Cows carrying the TT genotype tended to be calved earlier than CT heterozygotes (p = 0.069) and homozygote CC genotypes (p = 0.031). The TT genotype conferred a 24-day advantage over the CC genotypes. A positive tendency of the heterozygote CT genotype was observed on DO (Table 3), as cows with this genotype of *LEP* g.-963C>T had shorter periods between calving and pregnancy (p = 0.052). Repeated measurements also showed a positive tendency of the CT genotype on PR (p = 0.091) (Table 3). We did not find significant differences or notable tendencies among genotypes of *LEP* g.-963C>T in any other traits related to fertility.

In the c.357C>T mutation, we observed, that cows carrying the TT genotype had, in repeated measurement analysis, a tendency for longer CLI than the CT heterozygotes (p = 0.094). We confirmed a negative effect of the TT genotype on CLI at the 3rd parity (Supplementary Figure S2) in comparison to CT genotype (p = 0.024). Similarly, we observed a negative effect of the TT genotype on DO at the 2nd parity. Cows with the TT genotype showed the longest DO at the 2nd parity (Supplementary Figure S3), compared to CC or CT genotypes (p = 0.006 and p = 0.009, respectively). We observed a tendency for advantage of the CC genotype on PR during the 1st parity (p = 0.085).

Regarding the *TLR4* g.-226C>G polymorphism we observed an association of its genotypes with CLI. Firstly, we observed a tendency for shorter CLI in heterozygote cows during the 3rd parity (p = 0.059) than CC cows (Supplementary Figure S2). The repeated measurements analysis confirmed this association on a significant level (Table 3), where CG genotype presented shorter CLI than did CC genotype (p = 0.029). We also observed a tendency for associations of *TLR4* g.-226C>G with PR. Heterozygous cows showed higher PR at the 1st parity (p = 0.096) than cows carrying the GG genotype (Supplementary Figure S4).

We did not observe any effects of SNP c.777C>G in *CXCR1* in Czech Fleckvieh cattle. The repeated measurement analysis also failed to confirm any significant associations.

## DISCUSSION

### *In silico* analysis

The *LEP* c.357C>T is considered to be deleterious and in the association analysis we observed negative tendency of this SNP on CFI and PR at 1st parity. These tendencies describing the negative effect of TT genotype of the *LEP* c.357C>T were confirmed at a significant level in CLI (3rd parity) and DO (1st parity).

The SNP *CXCR1* c.777C>G is probably benign and this mutation has no effect on fertility traits in Czech Fleckvieh cattle. The in-silico analysis results are consistent with the observed associations and amend the knowledge about its function.

### Single nucleotide polymorphism association

These results demonstrate associations between fertility traits and the *LEP* and *TLR4* genes. These are the first associations presented for immune response genes and fertility in Czech Fleckvieh cattle. We present results for repeated measurements and observations by parity to show how the associations differed in cows of different lactations and stages of physiological developments. Although we note that for breeding, it is also important to describe the associations in general, for longer production period. The combination of the 1st three parities allowed us to present more complex associations and consider metabolic differences between young cows with incomplete growth and older cows on their 3rd parity. We would like to mention that in our experiment we found no association with the CFI trait in investigated SNP (Table 3, Supplementary Figure S1). We suppose that this trait is strongly affected by the farmer’s decision and the animal reproduction potential is better presented by DO.

### Leptin g.-963C>T

The g.-963C>T SNP was described to be associated with pregnancy rate at 100 days [3], as the CC genotype was associated with a decrease in the rate of successful pregnancies. This result is in accordance with our observations of PR. Furthermore, the TT genotype was associated with shorter CLI and DO periods [3], which is consistent with our results in Czech Fleckvieh. Additionally, we observed the longest DO interval in CC cows; therefore, in general we can conclude a positive effect of the T allele on studied traits. Conversely, we did not find any differences between genotypes on CLI intervals. Similarly, no association was described with studied fertility traits in Jersey cattle [4,18]. Liefers et al [2] described a negative effect of the T allele on the first observed estrus after parturition. Our results showed a positive effect of the TT genotype on AFC. This finding can suggest, that the first estrus may occur later, but when served, animals with the TT genotype have higher PR. Increased fertility of cows with the *T* allele could be due to higher dry matter intake, higher growth rate and better management of negative energy balance after calving, as pointed out by Liefers et al [2].

Jecminkova et al [26] also investigated the g.-963C>T mutation in a study of population diversity of Czech Fleckvieh cows hybridized with the Holstein breed and cows of the original Czech Fleckvieh breed. In this study, the hybridized cows showed a departure from H-W equilibrium with predominance of the CC genotype. In addition, other population diversity parameters indicated an indirect selection pressure toward the CC genotype through increasing milk yield. In accordance with Clempson et al [3], the g.-963C>T mutation might be involved in the genetic markers influencing cattle fertility.

### Leptin c.357C>T

In our experiment cows with the TT genotype always showed worse reproduction than the CT genotype cows. The TT cows presented the lowest PR at the 1st parity. Our finding is not in concordance with results in Jersey cows as was described by the shortest CLI and DO in TT genotype cows [18]. Results of the repeated measurements analysis show that the TT genotype cows had the tendency for longest CLI. This was compatible with the results from the lactation analysis, but was only significant at the 3rd parity (Supplementary Figure S2).

We also observed unfavorable effects of the TT genotype on DO where cows had 21 days longer interval than heterozygote cows. Conversely, Yazdani et al [27] found no association with DO and CLI. Szyda et al [28] described TT cows as having a 1.83 times higher risk of culling than CC cows. Their finding could be similar to our results, as the TT genotype was associated with decreased fertility and the decreased fertility is one of the most common reasons for culling.

### Toll-like receptor 4 c.-226C>G

The fertility can be disturbed by inflammation of reproduction organs, as they are, mostly after calving, sensitive to bacteria contamination. This contamination can be caused by *Escherichia coli* (*E. coli*), as the bacteria are present in feces. Therefore, it can invade the genital tract during and after parturition. *TLR4* is located on cell surfaces and recognizes lipopolysaccharide of gram-negative bacteria [8], and thus is mostly involved in the protection against its deleterious effects. Described in the udder, the *TLR4* expression strongly increases in infection caused by *Staphylococcus Aureus* and *E. coli* [29,30]. According to Herath et al [10], cows could maintain fertility by limiting the inflammatory response during the 1st week postpartum. As the GG cows had significantly higher expression of *TLR4* [7], their fertility could be compromised. The reason can be the over activation of an immune response that may break out immune equilibrium and lead to the injury of hosts [9]. In our study, the CG genotype was favorable for CLI and showed a nonsignificant tendency for higher PR. Further, heterozygotes that showed intermediate levels of expression [7] had the best fertility and, therefore, could provide the optimal level of antibodies against gram-negative bacteria. This could be demonstrated in shorter CLI, because this indicator shows that no problem with reproduction occurred between calving.

### Chemokine receptor of interleukin 8 C-X-C motif c.777C>G

There are numerous studies describing an association of the c.777C>G mutation with milk yield or udder health. In literature, the interleukin 8 level was connected to several fertility diseases, e.g. endometritis or cystic ovarian disease; therefore, we decided to investigate its receptor. Nevertheless, no association of this polymorphism with fertility traits has been detected in cattle. Galvao et al [16] studied the influence of this polymorphism on metritis, endometritis, and retained fetal membranes, but did not find any association. However, they conducted their study only on 350 cows that were raised on 23 farms. Therefore, non-systematic effects could be difficult to eliminate. The relationship between *CXCR1* and fertility index was investigated in Italian Holstein [31], where no differences between genotypes were observed but the study was conducted only on sires. However, a study investigating the relationship between genotype and tradition fertility traits is missing as well as a study conducted on non-Holstein breed.

Verbeke et al [17], reported that the GG genotype was associated with higher milk yield. Higher milk yield may be associated with decreased fertility owing to the negative correlation between milk production and fertility traits [32]. Conversely, there are numerous studies describing a positive effect of the G allele in GG genotypes on somatic cell score (SCS) [14,15] and clinical mastitis resistance [17,33]. However, some studies have found no differences in SCS associated with the *CXCR1* genotype [20].

Based on previously published studies, we would expect a higher resistance toward infection in *GG* genotype cows. But, it is important to mention that previous studies were carried out on Holstein cattle, therefore, the associations may differ. Unfortunately, there is no association study conducted on dual-purpose cattle breeds similar to Czech Fleckvieh to compare with our results. More studies of *CXCR1* are necessary to understand the context of fertility performance and reveal significant effects.

## CONCLUSION

We confirmed a positive effect of the TT genotype of *LEP* g.-963C>T on age at 1st calving, and a positive tendency of the CT genotype on DO and PR. We also showed novel, contradictory results with respect to the c.357C>T mutation in *LEP*. It is clear that *LEP* SNPs are linked to reproduction in Czech Fleckvieh cattle and could be effective markers for improving reproductive traits. We show that the *TLR4* g.-226C>G polymorphism could be associated with fertility traits. Heterozygous genotype showed significant advantages for CLI and tendency for higher PR. The *CXCR1* SNP did not show any significant associations.

## Figures and Tables

**Table 1:** Descriptive statistics for reproductive traits, milk yield and somatic cell count

Trait	Nuliparous1)	Lactation 11)	Lactation 21)	Lactation 31)
AFC (d)	865±82.0 (791)	-	-	-
CFI (d)	-	73±35.9 (769)	72±21.7 (767)	75±23.4 (572)
DO (d)	-	102±52.4 (765)	101±48.0 (717)	107±52.8 (525)
CLI (d)	-	390±53.9 (772)	388±50.8 (683)	-
PR (%)	-	52.1 (791)	55.8 (747)	59.0 (659)
Milk yield (kg)	-	6,750±1,586 (791)	7,607±1,691 (747)	7,486±2,217 (659)
SCS (test days)	-	2.77±1.6 (1,815)	3.27±1.8 (1,781)	3.67±1.8 (1,046)

AFC, age at 1st calving; CFI, calving to the 1st insemination; DO, days open; CLI, calving interval; PR, pregnancy after the 1st service; SCS, somatic cell score.

1) Values are presented as mean±standard deviation. Values in parentheses are the number of records analyzed.

**Table 2:** Observed genotypic frequencies and results of chi-square tests

Gene	SNP	Genotype (n)	Frequency observed	Allele	Frequency	χ^2^
*LEP*	g.-963C>T	CC (436)	0.64	C	0.79	7.79**
		CT (207)	0.30			
		TT (44)	0.06	T	0.22	
		Total (687)				
*LEP*	c.357C>T	CC(457)	0.58	C	0.77	1.38
		CT (290)	0.37			
		TT (36)	0.05	T	0.23	
		Total (783)				
*TLR4*	g.-226C>G	CC (265)	0.35	C	0.58	1.37
		CG (357)	0.46			
		GG (143)	0.19	G	0.42	
		Total (765)				
*CXCR1*	c.777C>G	CC (305)	0.40	C	0.62	0.66
		CG (352)	0.45			
		CC (115)	0.15	G	0.38	
		Total (772)				

SNP, single nucleotide polymorphism; *LEP*, leptin; *TLR4*, toll-like receptor 4; *CXCR1*, chemokine receptor of interleukin 8 C-X-C motif.

** p<0.01.

**Table 3:** SNP associations with fertility traits in repeated measurements analysis

Gene Genotype	Traits				

	AFC1)	CFI1)	DO1)	CLI1)	PR (%)2)
*LEP* g.-963C>T					
CC	860 (852.9, 868.3)^a^	69 (67.8, 72.0)	98 (93.5, 104.0)^A^	391 (385.5, 397.3)	52.1±2.2^A^
CT	858 (848.8, 868.4)^B^	68 (66.0, 70.7)	92 (87.1, 98.3)^A^	388 (381.7, 395.1)	58.6±2.7^A^
TT	836 (818.0, 854.7)^a, B^	71 (67.2, 75.8)	93 (84.6, 103.7)	389 (378.7, 401.2)	59.7±2.7
*LEP* c.357C>T					
CC	857 (850.0, 865.8)	69 (67.5, 71.6)	95 (90.1, 100.4)	390 (384.3, 395.9)	56.7±2.1
CT	860 (851.9, 869.3)	68 (66.8, 71.0)	96 (91.4, 102.2)	389 (383.9, 395.8)^A^	53.2±2.3
TT	854 (834.1, 875.4)	70 (65.9, 74.8)	103 (92.2, 115.4)	401 (390.0, 413.6)^A^	47.8±5.7
*TLR4* g.-226C>G					
CC	853 (844.4, 862.7)	69 (66.7, 71.3)	98 (92.3, 104.0)	393 (387.1, 400.0)^a^	52.3±2.5
CG	863 (855.8, 872.2)	68 (66.8, 71.0)	93 (88.7, 99.0)	387 (381.3, 393.0)^a^	58.1±2.2
GG	855 (844.2, 867.1)	70 (68.0, 73.2)	98 (91.9, 104.9)	391 (384.3, 398.2)	52.1±3.1
*CXCR1* c.777C>G					
CC	860 (851.4, 869.1)	69 (66.8, 71.2)	95 (90.3, 101.3)	389 (383.8, 395.9)	55.5±2.4
CG	860 (852.4, 869.5)	69 (67.3, 71.6)	94 (89.7, 100.3)	388 (383.0, 394.9)	55.8±2.2
GG	850 (838.7, 863.2)	69 (66.2, 71.9)	96 (90.1, 104.3)	393 (395.9, 401.9)	53.6±3.5

SNP, single nucleotide polymorphism; AFC, age at 1st calving; CFI, calving to the 1st insemination; DO, days open; CLI, calving interval; PR, pregnancy after the 1st service; *LEP*, leptin; *TLR4*, toll-like receptor 4; *CXCR1*, chemokine receptor of interleukin 8 C-X-C motif; SCS, somatic cell score; LSM, least squares mean; CI, confidence intervals.

1) LSM (95% CI).

2) LSM±standard error.

Values followed by the same superscript letters, within the same column of locus, indicate significant differences after Bonferroni adjustment between the genotypes at p≤0.05 (lowercase) or statistic tendencies for difference at p≤0.10 (uppercase).
