# Supplementary material for: Association of leptin, toll-like receptor 4, and chemokine receptor of interleukin 8 C-X-C motif single nucleotide polymorphisms with fertility traits in Czech Fleckvieh cattle
Source: Asian-Australas J Anim Sci. 2018 Apr 12;31(11):1721–8. doi: 10.5713/ajas.17.0900 (PMC6212755; doi:10.5713/ajas.17.0900)
Supplement: Supplementary file 1 [file ajas-31-11-1721-supplementary.pdf]

Supplementary Table S1 *In silico* analysis of *LEP* and *CXCR1* SNP using PANTER, PROVEAN and I-Mutant2.0

Amino acid change	PANTER prediction	PROVEAN prediction	PROVEAN score	Reliability index	Difference in energy after substitution (Kcal/mol)	Stability prediction
LEP c.357C>T Alanine > Valine	Possibly damaging	Neutral	-0.991	1	0.63	Increase
CXCR1 c.777C>G Glutamine > Histidine	Probably benign	Neutral	4.387	4	-0.44	Decrease

Supplementary Figure S1. Relationship between studied genotypes and CFI according to the parity number

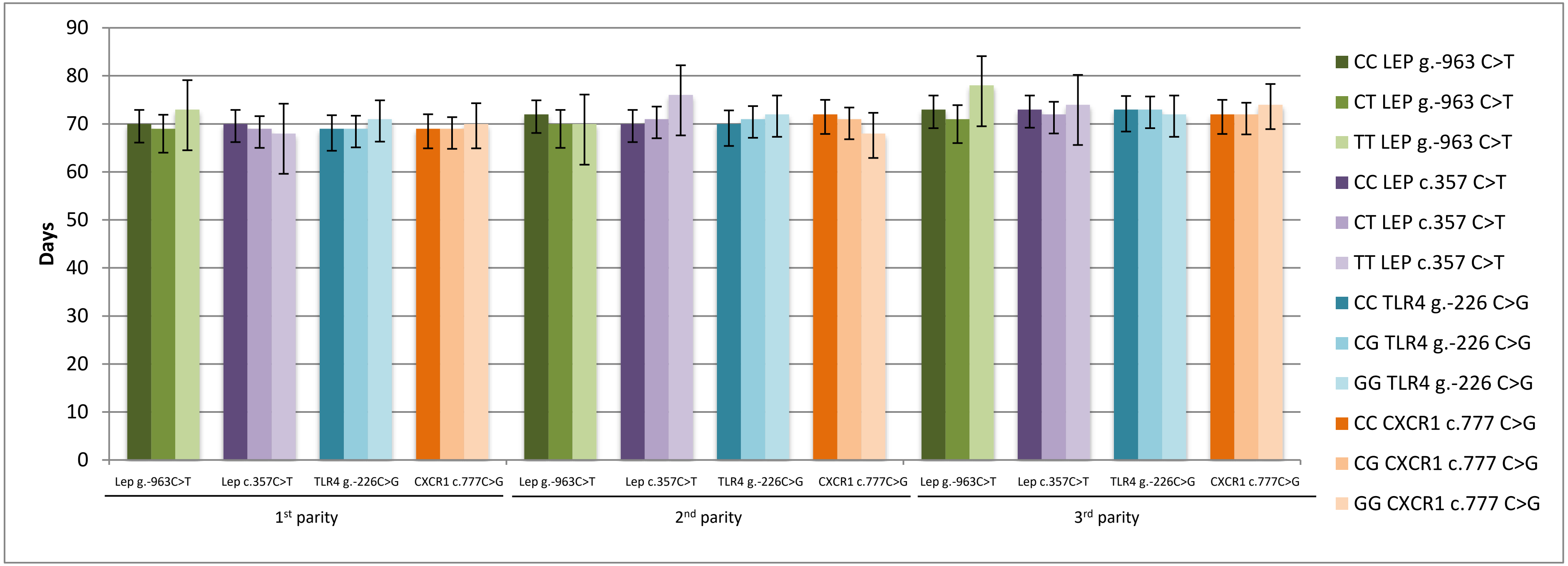

LSM with 95 % confidence interval.

Supplementary Figure S2. Relationship between studied genotypes and CLI according to the parity number

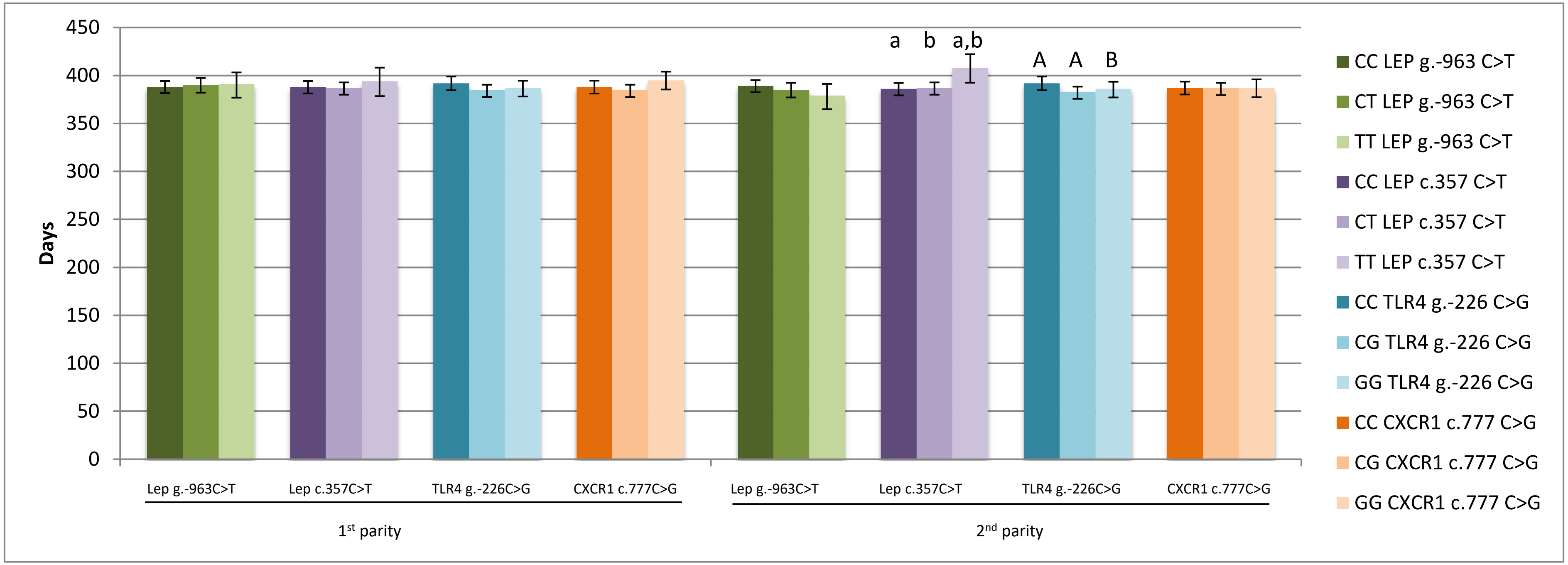

Values followed by the same superscript letters, within the same column of locus, indicate significant differences after Bonferroni adjustment between the genotypes at $P \leq 0.05$ (lowercase) or statistic tendencies for difference at $P \leq 0.10$ (uppercase).

LSM with 95 % confidence interval.

Supplementary Figure S3. Relationship between studied genotypes and DO according to the parity number

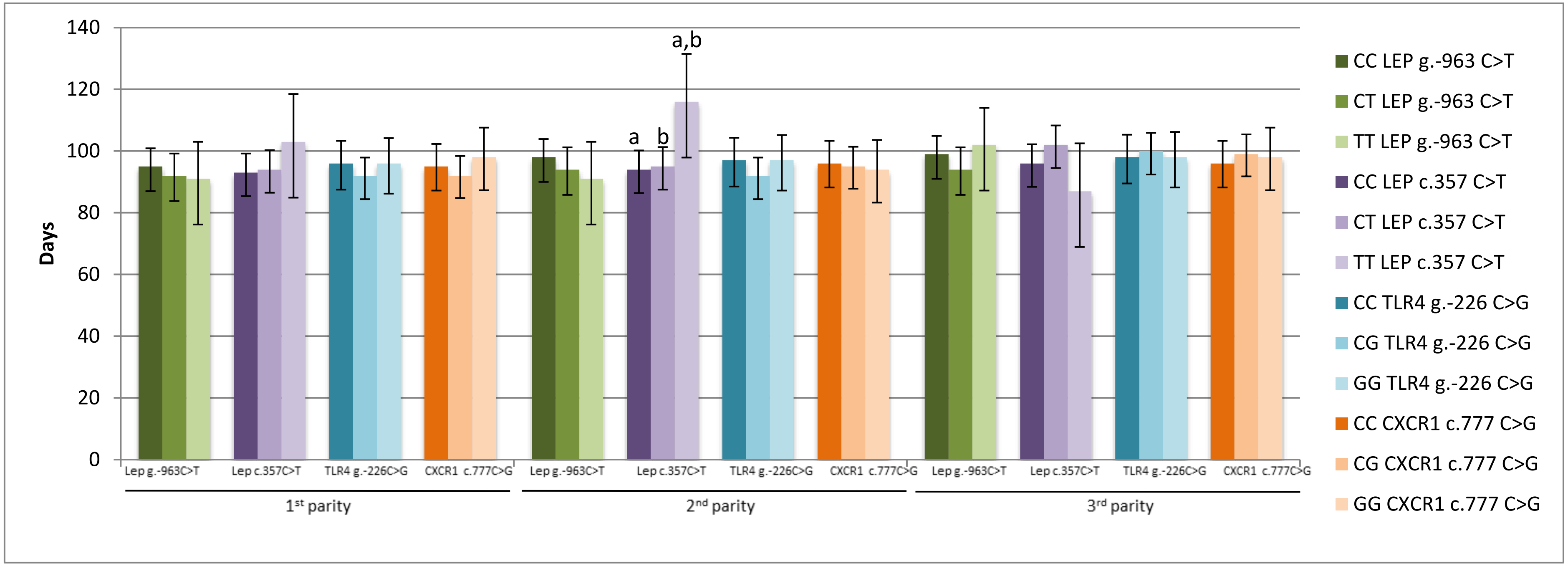

Values followed by the same superscript letters, within the same column of locus, indicate significant differences after Bonferroni adjustment between the genotypes at $P \leq 0.05$.

LSM with 95 % confidence interval

Supplementary Figure S4. Relationship between studied genotypes and PR according to the parity number

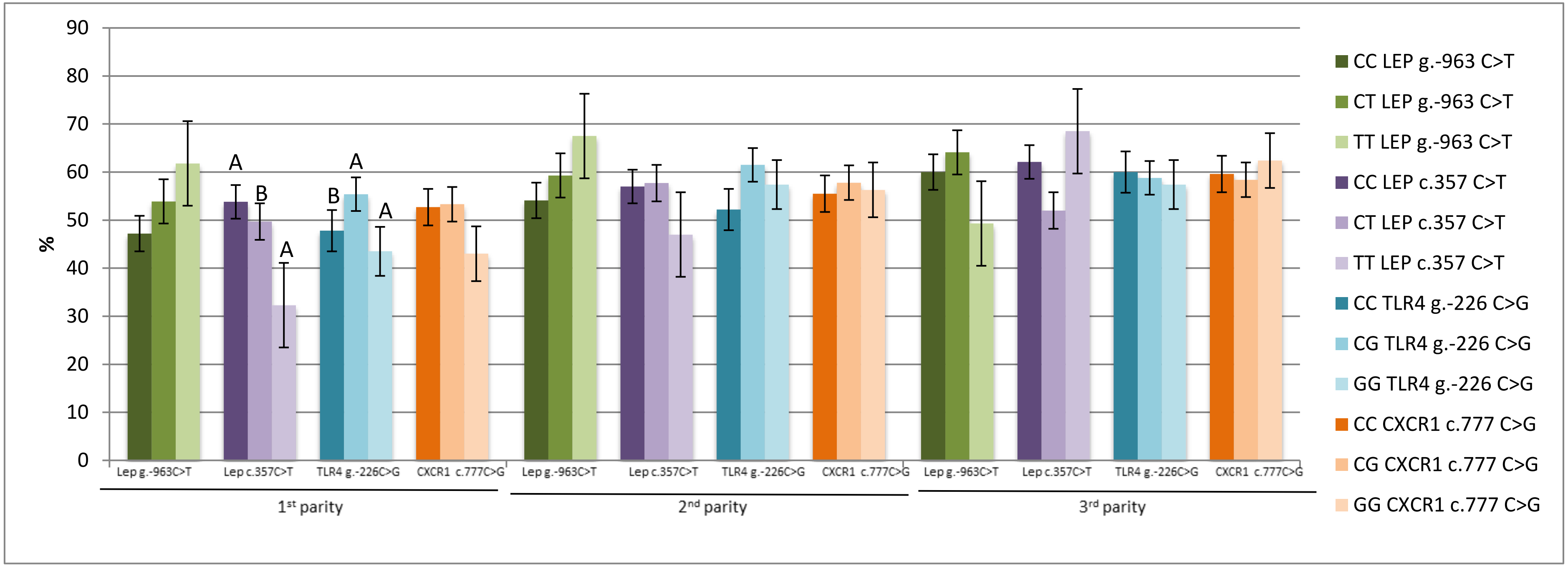

Values followed by the same superscript letters, within the same column of locus, indicate statistic tendencies for difference at $P \leq 0.10$.

LSM ± standard error
